# Window Area and Development Drive Spatial Variation in Bird-Window Collisions in an Urban Landscape

**DOI:** 10.1371/journal.pone.0053371

**Published:** 2013-01-09

**Authors:** Stephen B. Hager, Bradley J. Cosentino, Kelly J. McKay, Cathleen Monson, Walt Zuurdeeg, Brian Blevins

**Affiliations:** 1 Department of Biology, Augustana College, Rock Island, Illinois, United States of America; 2 Department of Biology, Hobart and William Smith Colleges, Geneva, New York, United States of America; 3 BioEco Research and Monitoring Center, Hampton, Illinois, United States of America; 4 Reynolds, Illinois, United States of America; 5 Davenport, Iowa, United States of America; 6 Davenport, Iowa, United States of America; University of Western Ontario, Canada

## Abstract

Collisions with windows are an important human-related threat to birds in urban landscapes. However, the proximate drivers of collisions are not well understood, and no study has examined spatial variation in mortality in an urban setting. We hypothesized that the number of fatalities at buildings varies with window area and habitat features that influence avian community structure. In 2010 we documented bird-window collisions (BWCs) and characterized avian community structure at 20 buildings in an urban landscape in northwestern Illinois, USA. For each building and season, we conducted 21 daily surveys for carcasses and nine point count surveys to estimate relative abundance, richness, and diversity. Our sampling design was informed by experimentally estimated carcass persistence times and detection probabilities. We used linear and generalized linear mixed models to evaluate how habitat features influenced community structure and how mortality was affected by window area and factors that correlated with community structure. The most-supported model was consistent for all community indices and included effects of season, development, and distance to vegetated lots. BWCs were related positively to window area and negatively to development. We documented mortalities for 16/72 (22%) species (34 total carcasses) recorded at buildings, and BWCs were greater for juveniles than adults. Based on the most-supported model of BWCs, the median number of annual predicted fatalities at study buildings was 3 (range = 0–52). These results suggest that patchily distributed environmental resources and levels of window area in buildings create spatial variation in BWCs within and among urban areas. Current mortality estimates place little emphasis on spatial variation, which precludes a fundamental understanding of the issue. To focus conservation efforts, we illustrate how knowledge of the structural and environmental factors that influence bird-window collisions can be used to predict fatalities in the broader landscape.

## Introduction

Urbanization fundamentally changes ecosystem function and structure and has profound effects on wildlife populations. Urban development alters avian community structure by reducing overall richness and diversity of species and increasing densities of synanthropic species [1]–[3]. Birds that reside in urban settings face numerous human-related threats to survival, including mortality from bird-window collisions (BWCs) [4]. Window glass is an invisible barrier to birds, and collisions occur as birds attempt to fly through what appear to be reflections of open space and vegetation [5]–[6]. BWCs are suspected to be ubiquitous across the urban landscape, and current estimates assert that 1–10 birds die from a window strike at every building each year in the United States, including structures that range from small houses to large skyscrapers [7].

Understanding the magnitude and drivers of BWCs is important because urbanization is accelerating faster than human population growth [8], and knowledge of how the urban environment affects bird survival is needed for conservation and management. For example, mortality from power line collisions increased extinction risk for Ludwig’s Bustard (*Neotis ludwigii*) in South Africa [9], and mortality from window strikes may be affecting birds in similar ways [10], but see [11]. Furthermore, cities display complex spatial patterns of development, which is affected by historic landscape configurations and current social, economic, and political climates [8], [12]–[13]. This results in patchily distributed resources and developed space creating the expectation that the magnitude and species affected by window collisions should vary across the landscape.

Environmental resources have been hypothesized to be a primary driver of BWCs. Quality vegetation and artificial feeding stations increase bird density by providing food and shelter [14]–[17], and the number of fatalities is predicted to be proportional to species abundance [5]. For example, houses maintaining feeder stations during the winter attract high numbers of sparrows and finches, which account for more strike-related deaths than other species [5]–[18]. However, some species with low abundance die at high rates (e.g., Ovenbird) and several abundant species are not susceptible to collisions (e.g., House Sparrow) [19]–[20]. It remains equivocal that the environmental resources hypothesis explains BWCs across the urban matrix because most research has been restricted by low replication of buildings and buildings with known mortalities [4]. If the environmental resources hypothesis is supported, the magnitude of fatalities should be related to factors that increase bird density and richness.

The amount of sheet glass in buildings is also hypothesized to influence BWCs. Support for the window area hypothesis comes from localized studies reporting high mortality at large commercial buildings [19]–[23]. However, urban landscapes have variable patterns of development that often include a core commercial district composed of many large buildings and clusters of mixed-use development where office space and residential areas coexist [24]. Currently, ∼20% of the total building area of commercial space in the United States may be found in suburban areas [24]. This suggests that fatalities should be highest at the largest buildings in the urban matrix, whereas small residential buildings with low window area should pose the lowest risk. However, we are unaware of studies that have evaluated the relative magnitude of BWCs among buildings of varying size.

We characterized bird community structure and documented the number of BWCs (hereafter synonymous with mortality) at 20 buildings of variable size in an urban landscape in northwestern Illinois, USA. Our primary objective was to test the environmental resources and window area hypotheses. We first evaluated how bird abundance, richness, and diversity were related to environmental factors. We then tested whether BWCs were related to environmental factors that influence community structure and to building window area. The potential for imperfect detection of carcasses was evaluated experimentally by estimating carcass persistence times [25] and detection probabilities by field workers, and we used those estimates to design a sampling scheme that minimized detection bias. We illustrate how knowledge of the structural and environmental factors that create spatial variation in BWCs can be used to focus conservation efforts in high-risk settings. The utility of these factors, rather than direct estimates of bird abundance and diversity, is that they are readily available in print and digital media to parties interested in predicting BWCs, but whose expertise lies outside of ornithology.

## Methods

### Ethics

Protected species were not sampled and we followed the recommendations of Fair et al. [26] in reducing impacts to birds resulting from investigator presence during point count surveys. Carcasses collected during field surveys and those used in experiments were salvaged under state Scientific Permit (#NH11.0313), Illinois Department of Natural Resources, and federal Salvage Permit (#MB08907A-0), U.S. Fish and Wildlife Service. We consulted Fair et al. [26] for recommendations related to collecting procedures of bird carcasses.

### Study Buildings

We conducted the study at 20 buildings in Rock Island and Moline in northwestern Illinois. This 9,330-ha urban area is bordered to the north and west by the Mississippi River and to the south by the Rock River, and it is located in the Dissected Till Plains Physiographic Area [27]. We used stratified random sampling in ArcGIS (ESRI, Redlands, California, USA) to select 20 study points distributed among four land cover categories: (1) High Urban Density (>50% covered with structures), (2) Low/Medium Urban Density (up to 50% covered with structures), (3) Urban Open Space (parks, golf courses, cemeteries, and other grassland-like cover within urban and built-up areas), and (4) Forested Land and Floodplain Forest (undeveloped land that occasionally includes buildings) [28]. A stratified design ensured selection of a sample of buildings with sufficient variation in land cover. We obtained permission to use buildings on private and public land closest to each point. Two property owners denied permission, and we obtained permission to access the next closest building. Buildings ranged in size from small single-family residential to small commercial (110–700 m^2^ floor area). However, large commercial buildings were less common in our study area and were not represented in the initial sample of 20 points. Therefore, we opportunistically selected five large buildings (3750–14950 m^2^ floor area) within Urban Open Space (N = 2), High Density (N = 2), and Forest (N = 1), which replaced five randomly selected small buildings in the same land cover categories. Median distance between buildings was 917 m (range 356–1976 m).

### Point Count Surveys

We used 50-m radius point counts of 5-min duration to characterize the avian community at each study building in 2010 [29]–[30]. Three surveys were completed in each of three one-week sampling periods in each season for a total of nine counts/season (Table S2). Each point count location was ≤50 m from the edge of a study building and >50 m from public roadways. All sites were surveyed on a single survey day, and surveys began at sunrise and were completed within 5 h. We assigned each building a number from 1–20, and we randomly selected the starting location each day. Subsequent locations were sampled in numerical order. Varying survey order decreased the likelihood of missing species that may vary in daily activity [31]. Point counts were conducted by one of us in a season (BB in spring, KJM in fall, and SBH in winter and summer) and during favorable weather conditions [31]. Seasonal variation in community structure may have been affected by observer differences, but scheduling issues precluded sampling by just one person for all seasons.

We identified and counted all birds seen and heard during each survey. For each building and season, we calculated abundance as the sum of the maximum number of individuals counted within each of the three sampling weeks [30]. Species richness was the total number of species observed in each season, and diversity was measured using the Shannon diversity index [32]. The following species were excluded from analyses: birds flying over the site, migratory flocks, waterfowl, raptors, and species seen on <2 surveys/season [33]. Scientific names of birds documented during point counts and listed in the text are found in Table S4.

### Carcass Surveys

For each building and season, we completed 21 daily carcass surveys during three weekly sampling periods concurrent with point count surveys (Table S2). During each survey, a trained observer walked a complete transect around the building and searched for bird carcasses within a 2-m buffer from the building’s edge [20]. A bird carcass consisted of a full body, partial carcass, or feather piles [25]. Observers actively searched through woody vegetation located within the transect because birds may fall into a shrub after a window collision. All surveys in the winter coincided with post-snowfall events and, thus, detection of carcasses was high due to the hole created in the otherwise unbroken snow layer from a falling bird. Surveys in the fall were completed before the first hard freeze and extensive leaf drop by deciduous trees and shrubs.

Carcasses and corresponding identification tags were placed in food-grade, zip-lock plastic bags. We stored carcasses on ice until placement in a freezer <6 hours after collection, and we identified carcasses to species in the laboratory. Partial carcasses were categorized as “unidentified” if species-important anatomical features were missing due to scavenging. Birds were classified as adult or juvenile based on plumage and degree of cranial pneumatization [34].

Surveying a subsample of days limited a full representation of carcasses that might have arisen from window strikes. However, we attempted to reduce this bias by completing surveys during times of important bird activities (Table S2) [35]. We also minimized bias associated with imperfect detection of carcasses by using a sampling design that was informed by estimates of carcass persistence before scavenging [25] and carcass detection probability by field workers (Text S1, Table S1, Figure S1). Specifically, carcass survival in relation to scavengers was estimated for each building-season combination in 2010 [25]. Using an exponential model of survival time, we found that carcasses generally persist at buildings for ≥3.5 days. Detection of carcasses in the field was related to carcass observability and field worker. However, the overall average detection probability was very high (0.88, SE = 0.01; Figure S1). Given the long persistence time and high detection probability of carcasses, the likelihood of not detecting a carcass using daily surveys was low. Carcasses found on day 1 of all survey periods were collected, but they were not included in analyses. Because scavenging pressure varied among buildings [25], including fatalities from surveys on day one may have introduced detection bias.

### Environmental and Building Covariates

Land cover attributes were digitized for study buildings and point counts from a Bing Map high-resolution aerial photograph in ArcGIS taken during the growing season of 2010 (ESRI, Redlands, California, USA). We characterized land cover in a 50-m buffer zone extending from exterior walls of buildings and from the center point of count circles for bird surveys [36]. We considered quantifying habitat in varying buffer distances, but land cover in our 50-m buffer zones was highly correlated with land cover at larger scales (r >0.80 for all 50-m intervals up to 250 m) [28]. Percent area was estimated for (1) canopy (canopy cover of trees and large shrubs), (2) exposed habitat (grass/lawn, landscaped ground cover, and open water), (3) structures (buildings), and (4) pavement (roadways, sidewalks, and parking lots). A 50-m buffer captured detailed and ecologically relevant attributes related to the distribution and activity of urban birds at a local scale, e.g., [15]. Moreover, urban bird diversity and abundance consistently correlates positively with vegetated features and negatively with impervious surfaces, e.g., [37]. Thus, we combined digitized land cover categories into two broader classes: undeveloped (canopy and exposed habitat) and developed (structures and pavement), e.g., [38]. Only proportion of developed land was used in analyses. Because birds also respond to landscape-scale feature such as vegetated patches within the urban matrix e.g., [16]–[39], we calculated the average distance between point count locations and all vegetated patches >0.5 ha in the study area [39]. A taped rule was used to measure the area of windows in each building.

Buildings were classified as having feeder stations if at least one active feeder was visible within 50-m of a building’s edge. We used this classification for two reasons. First, identifying feeders within 50 m is consistent with our test of the environmental resources hypothesis, which predicts that bird-friendly resources that increase bird density will influence collision fatalities. Second, birds that visit feeders appear to strike windows at a nearby building during a panic flight, or the explosive flight away from the feeder in response to the sudden appearance of a potential predator [5]. Fatal collisions occur at distances ≤10 m, although mortality is highest at 10 m [40]. It is thought that birds flushed by a predator from feeders at 10 m gain enough momentum (via high flight speed) to strike a window resulting in fatal injury [40]. Using this information, we assumed that birds flying toward a window from >10 m also have comparable flight speeds that would make them vulnerable to dying from a window strike. Thus, birds flushed from a feeder at <50 m of a building’s edge should be vulnerable to BWC’s. Table S3 lists minimum distance to feeders for study buildings.

### Data Analysis

We used linear and generalized linear mixed models (GLMM) to evaluate how bird abundance, species richness, and diversity were related to environmental factors. A random intercept was estimated for each building. A Poisson distribution with a log link function was specified for abundance and species richness, and a Gaussian distribution with an identity link function was specified for diversity. We constructed models that included different combinations of environmental factors as predictor variables. Environmental factors included presence of feeder station (F), proportion of developed land (D), and average distance to vegetated patches (I). We assumed *a priori* that the response variables varied among seasons, so we included an effect of season (S) in each model. We parameterized 21 models, and the candidate set was the same for each response variable. The first 8 models included an effect of season alone (S) and additive effects of each factor (S+F, S+D, S+I, S+F+D, S+F+I, S+D+I, S+F+D+I). We also evaluated the support of 12 models that included interaction effects between season and each environmental factor (S+F+S*F, S+D+S*D, S+I+S*I, S+F+S*F+D, S+F+S*F+I, S+D+S*D+F, S+D+S*D+I, S+I+S*I+F, S+I+S*I+D, S+F+S*F+D+I, S+D+S*D+F+I, S+I+S*I+F+D). Finally, we included an intercept-only model.

We used GLMMs to evaluate whether the number of carcasses at each building depended on environmental factors (F, D, I) and building window area (W). A random intercept was estimated for each building, and we specified a Poisson distribution with a log link function for the response variable. We evaluated the support of 11 models that included different combinations of covariates. The first 10 models included individual factors (W, F, D, I) and additive effects of each factor (W+F, W+D, W+I, F+D, F+I, D+I). We also included an intercept-only model. The natural logarithm of window area was used to improve linearity. We did not include an effect of season in models because of the relatively low number of collisions observed during each season.

For both bird community structure and BWCs, we used the Akaike Information Criterion corrected for small sample size (AIC_C_) to evaluate the relative support of each model in each candidate set [41]. We calculated the difference between AIC_C_ of each model and AIC_C_ of the most-supported model (Δ AIC_C_), and we considered models to have competitive support when Δ AIC_C_ ≤2. We also calculated Akaike weights (*w*_i_) for each model. The lmer function in package lme4 [42] in program R [43] was used to fit all models.

### Predicted Annual Fatalities

We created a map of annual predicted fatalities to visualize spatial variation in collision risk in our study area. We intended to predict annual fatalities at all buildings in the study area using factors from our most-supported model of BWCs (i.e., window area and proportion of development; see Results). However, information on window area and proportion of development was not readily available at all buildings. Instead, we used surrogate features, which were easily obtained from local and federal agencies. Due to their high correlations, we used floorspace (i.e., the total usable living and office space contained within a building) as a surrogate for window area (*r = *0.99) and impervious development measured from the National Land Cover Database (NLCD) [44] as a surrogate for development (*r = *0.88; Table S3). We fit a generalized linear mixed model (i.e., the ‘surrogate’ model) of observed mortalities using floorspace and NLCD development as predictors.

We obtained floorspace for 1,956 buildings (hereafter referred to as 'model buildings') in Rock Island and Moline from the Rock Island County GIS Department. Floorspace was restricted to parcels containing single buildings only. The City of Moline GIS Department provided digitized building footprints for Moline (n = 996) and we manually digitized footprints for Rock Island buildings (n = 960). Proportion of NLCD development within a 50 m buffer surrounding each building footprint was calculated using ArcGIS (ESRI, Redlands, California, USA). We used beta coefficients from the surrogate model to predict number of fatalities at model buildings. Predicted fatalities at model buildings were then spatially interpolated for the study area using ordinary kriging in ArcGIS (ESRI, Redlands, California, USA).

## Results

### Avian Community Structure

**Figure 1:**
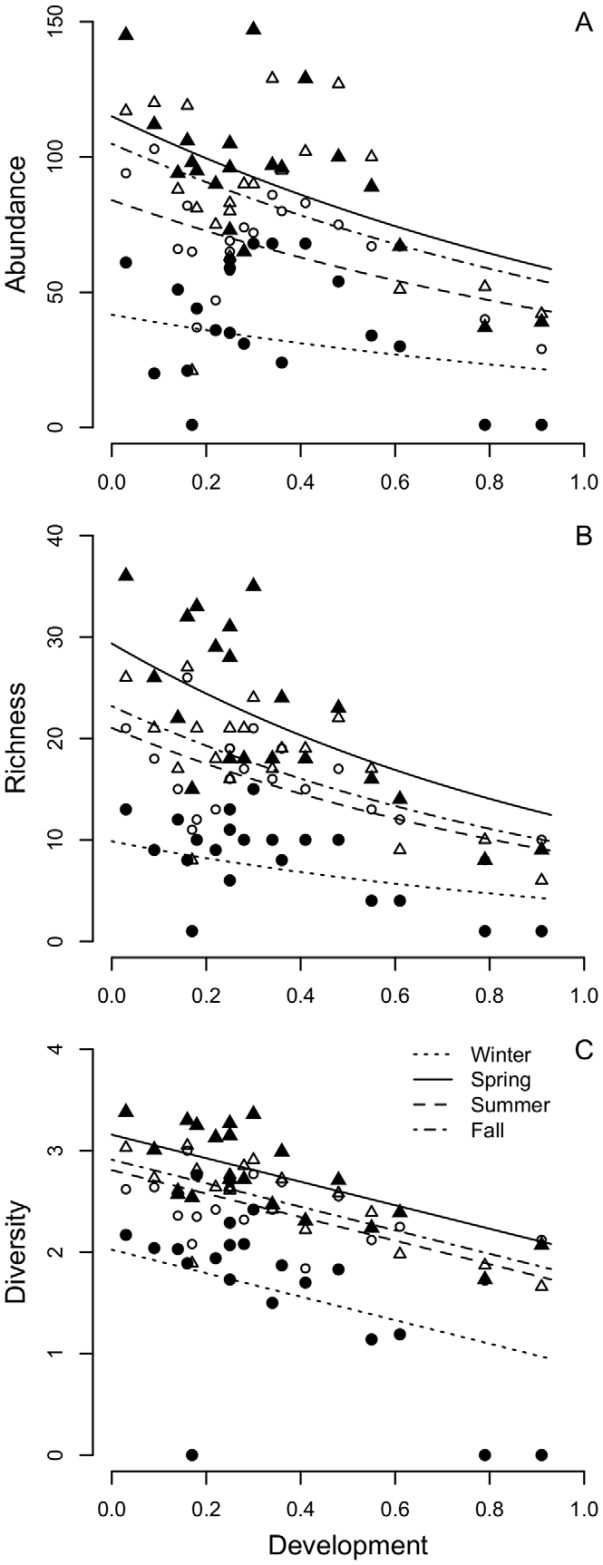
Effect of proportion of developed land on avian community structure. Relationships of avian (A) abundance, (B) richness, and (C) diversity are characterized for winter (closed circles), spring (closed triangles), summer (open circles), and fall (open triangles). Best-fit lines are indicated for each season and are based on parameter estimates from the most-supported models of each response variable (see Table 1).

**Figure 2:**
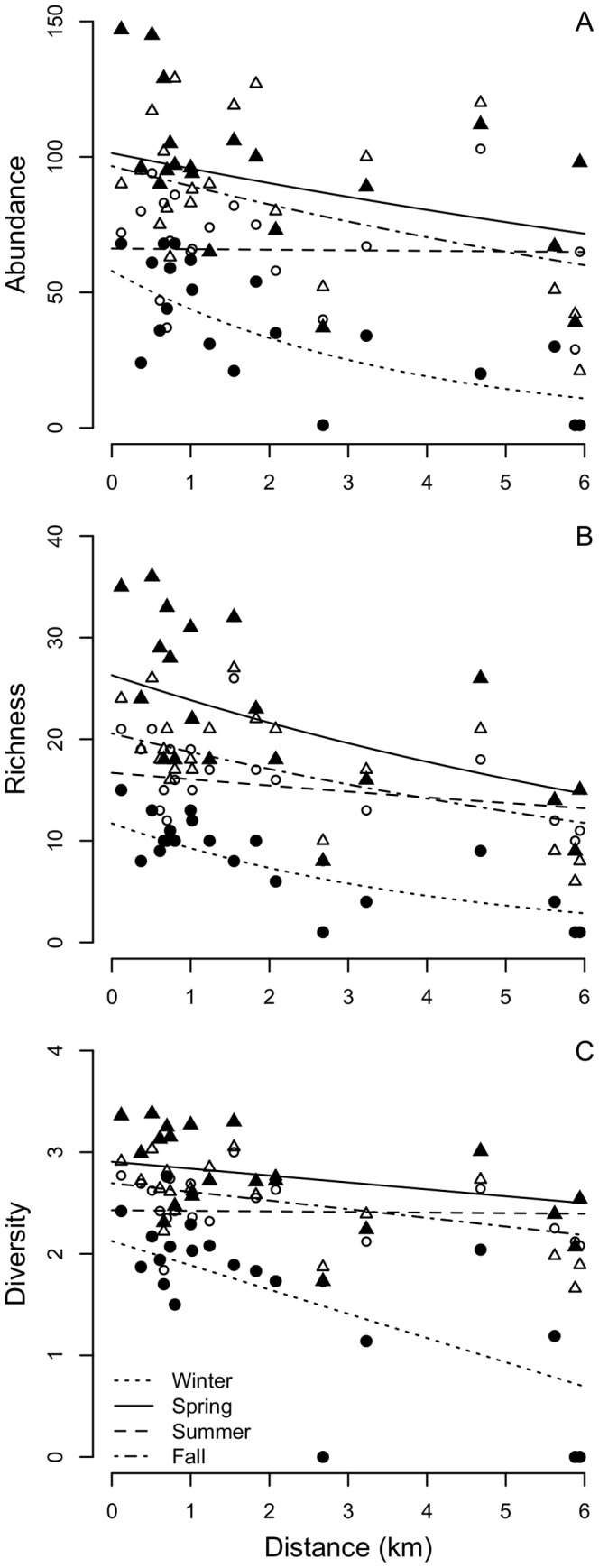
Effect of distance to vegetated lots on avian community structure. Relationships of avian (A) abundance, (B) richness, and (C) diversity are characterized for winter (closed circles), spring (closed triangles), summer (open circles), and fall (open triangles). Best-fit lines are indicated for each season and are based on parameter estimates from the most-supported models of each response variable (see Table 1).

We documented 23 species in winter, 57 in spring, 38 in summer, and 49 in the fall for an annual total of 72 species among study buildings (Table S4). The most-supported model was consistent for all community indices and included effects of season, development, and distance to vegetated lots (Table 1). In general, abundance, richness, and diversity were greatest in spring, fall, and summer, and lowest in winter (Fig. 1, 2). Abundance was related negatively to development (Fig. 1A; beta estimate from most-supported model = −0.73, SE = 0.25) and distance (Fig. 2A; beta estimate from model with season and distance only = −0.11, SE = 0.03). However, the effect of distance depended season. The negative relationship between abundance and distance was strongest in winter and weaker in spring and fall (Fig. 2A). There was no relationship between abundance and distance in summer. There was competitive support for a model that included a positive effect of feeder presence on abundance (Table 1; beta estimate from most-supported model with feeder presence = 0.16, SE = 0.11). The House Sparrow was the most abundant species within and among seasons and at buildings maintaining feeders (Table S4). Other relatively abundant species included American Robin, American Goldfinch, Black-capped Chickadee, and European Starling.

**Table 1:** Most-supported models of avian abundance, richness, and diversity at 20 buildings in 2010 in Illinois, USA.

Avian Community	Model^a^	ΔAIC_C_	ω*_i_*	*L*	K
Abundance	S+I+S*I+D	0.00	0.54	−157.77	10
	S+I+S*I+D+F	0.73	0.38	−156.79	11
	S+I+S*I	4.65	0.05	−161.40	9
Richness	S+I+S*I+D	0.00	0.49	−29.29	10
	S+D+I	1.99	0.18	−34.10	7
	S+I+S*I+F+D	2.09	0.17	−28.98	11
	S+F+D+I	3.86	0.07	−33.80	8
	S+D+S* D+I	4.34	0.06	−31.46	10
Diversity	S+I+S*I+D	0.00	0.42	−26.85	10
	S+D+S*D	1.24	0.23	−28.78	9
	S+D+S*D+I	1.46	0.20	−27.58	10
	S+I+S*I+F+D	3.60	0.07	−27.30	11

Summary includes the relative difference between model AIC_C_ and the best model (ΔAIC_C_), Akaike weights (ω*_i_*), log-likelihood (*L*), and number of parameters (K). Only models with ΔAIC_C_ ≤5 are included.

a Main effects include season (S), feeder station presence (F), proportion of development (D), and average distance to closest vegetated patches (I).

Richness was related negatively to development (Fig. 1B; beta estimate from most-supported model = −0.92, SE = 0.20). Richness was also related negatively to distance (beta estimate from model with season and distance only = −0.14, SE = 0.03), but the effect of distance on richness depended on season. The relationship between richness and distance was weaker in summer than winter, spring, and fall (Fig. 2B).

Diversity was related negatively to development (Fig. 1C; beta estimate from most-supported model = −1.16, SE = 0.29) and distance (beta estimate from model with season and distance only = −0.16, SE = 0.04), but the effect of distance on diversity depended on season. The negative effect of distance on diversity was considerably stronger in winter than other seasons, and there was no relationship in summer (Fig. 2C). Interestingly, there was competitive support for an interaction effect between season and development, in which the negative effect of development on diversity was stronger in winter than other seasons (Fig. 2C).

### Carcasses at Buildings

Overall, we collected 46 carcasses resulting from BWCs, and BWCs were observed at 50% of study buildings. Of the 46 total carcasses collected, 34 carcasses were located during days 2–7 of survey periods and retained for data analyses. Only passerine and near passerine species (N = 16) were collected and these tended to be low to moderately abundant or were never detected during point counts (Table 2, Table S4). Abundant birds that did not die included the House Sparrow, American Goldfinch, and Black-capped Chickadee. Post-fledgling individuals represented 81% of the BWCs in summer and fall (Table 2). Mortality was observed at 50% of buildings that maintained feeder stations (n = 8), and only half of the species that died at these sites are known to visit feeders (Table S4).

**Table 2:** List of carcass species (N = 16) collected at 20 study buildings for each season in 2010 in Illinois, USA.

Species	Winter	Spring	Summer	Fall
Mourning Dove			1(1)	
Yellow-bellied Sapsucker				1(1)
Downy Woodpecker			1(1)	
Blue Jay				1(1)
Swainson's Thrush		1(0)		
Hermit Thrush				2(2)
American Robin			2(2)	1^a^
Gray Catbird		1(0)		
European Starling				1(0)
Cedar Waxwing			1(0)	3(2)
Common Yellowthroat				1(0)
White-throated Sparrow				2(2)
Dark-eyed Junco	1(0)			
Northern Cardinal	1(0)		1(0)	
Indigo Bunting		1(0)		
Common Grackle			2(2)	
Unidentified^a^	3	4		2
Total Individuals	5(0)	7(0)	8(6)	14(8)

Numbers in parentheses represent a count of hatch-year individuals of each season’s total.

a Carcass(es) partially scavenged and age-related features were not present.

**Figure 3:**
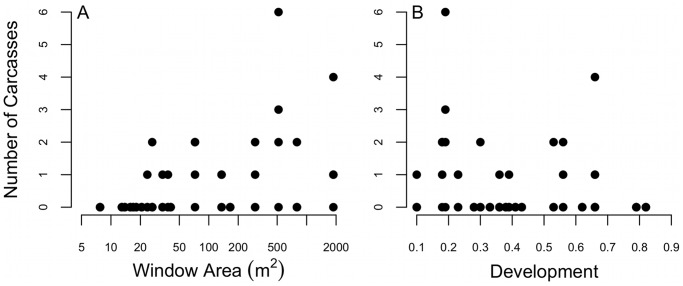
Factors driving bird-window collisions. The most-supported model explaining mortality included the effects of (A) window area and (B) development (% impervious surfaces) (see Table 3).

The most-supported model of collision mortality included the effects of building window area and proportion of developed land (Table 3). The number of fatalities was related positively to window area (Fig. 3A; beta estimate from top model = 0.83, SE = 0.14) and negatively to proportion of developed land (Fig. 3B; beta estimate from the model = −4.32, SE = 0.98). Both relationships resembled space-filling distributions, in which window area and development set upper bounds on the number of carcasses [45]. We observed no fatalities at buildings with <22 m^2^ of sheet glass or constructed in >66% development (Fig. 3).

**Table 3:** Most-supported models of avian mortality resulting from window collisions at 20 buildings in Illinois, USA, 2010.

Model^a^	ΔAIC_C_	ω*_i_*	*L*	K
W+D	0.00	0.999	−29.35	4
W+I	10.59	0.005	−34.64	4
W	11.49	0.003	−36.20	3
W+F	12.69	0.002	−35.69	4
D+F	22.99	0.000	−39.33	4

Summary includes the relative difference between model AIC_C_ and the best model (ΔAIC_C_), Akaike weights (ω*_i_*), log-likelihood (*L*), and number of parameters (K). The top five most-supported models are included.

a Main effects include window area (W), proportion development (D), and average distance to closest vegetated patches (I).

**Figure 4:**
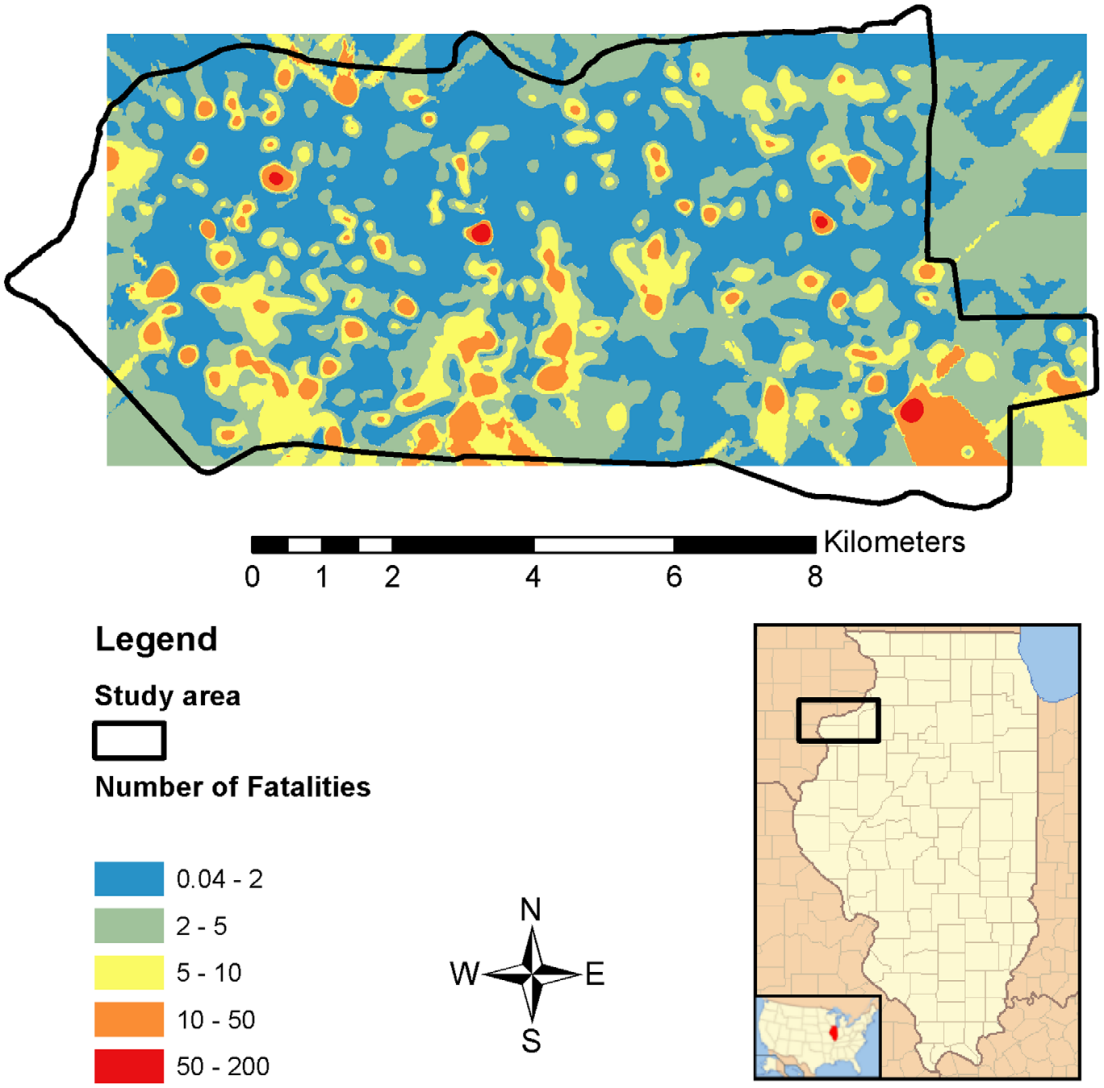
Predicted annual fatalities for the study area in Illinois, USA. Predicted fatalities were spatially interpolated from 1,956 model buildings using ordinary kriging.

The median number of predicted annual fatalities at study buildings based on (a) factors from the most supported model was 2.6 (range = 0.3–52.1) and (b) surrogate factors (i.e., floorspace and NLCD development) was 2.4 (range = 0.1–38.4; Table S3). The median predicted collision fatalities at 1,956 model buildings was 1.3 (range = 0.04–200.7). Spatially interpolated predicted fatalities at model buildings depict several small patches of high mortality where large buildings and low development coexisted, many small to large areas of moderate mortality, and low BWCs in the majority of the landscape (Fig. 4).

## Discussion

We assessed BWCs at buildings of various sizes and in a mix of habitats across an urban landscape. To our knowledge, the results reported here represent the most precise estimates of collision mortality to date, which were derived from a random sample of study sites and using a sampling protocol that reduced bias associated with imperfect detection. We found that BWCs were correlated positively to window area and negatively to development, which together created strong spatial variation in the number of fatalities. Previous estimates place a relatively constant and wide-ranging mortality at all buildings, e.g., 1–10 fatalities/building/year [7]. However, applying the drivers of BWCs to annual mortality estimates suggests that each building in the landscape has its own mortality ‘signature’. Furthermore, multiyear local studies indicate that this signature value varies little among years, e.g., [20]. Thus, knowing of the drivers of BWCs allows one to predict the magnitude of mortality for each building across the landscape, which is fundamental to conservation efforts aimed at reducing collision-related impacts.

The environmental resources hypothesis predicts that biotic resources that increase bird density and diversity will affect BWCs. We tested this hypothesis for each season of a year. Our results demonstrated that birds responded positively at sites with low levels of development and close to forested patches. These biotic factors are known to correlate with abundance and richness of breeding and winter residents [33], [36]–[37]. Migrating birds respond in similar ways, and vegetated lots ≥1 ha are valuable stopover locations during migration [38], [46]–[48].

Furthermore, we found that BWCs were affected by the proportion of development in the immediate vicinity of a building, but not by distance to vegetated patches. The influence of distance on community indices depended on season and, generally, the effect was greater in winter than non-winter. Indeed, winter mortality from window collisions was observed at only two buildings close to vegetated patches. Few carcasses were observed throughout the winter in general, which is consistent with other studies [20], [21]–[23]. These results suggest that BWCs are primarily affected by environmental resources at small scales.

The window area hypothesis predicts that collision mortality will be proportional to the amount of sheet glass installed in the exterior walls of a building, and our results supported this prediction. Windows are considered invisible barriers to birds in flight [7]. However, the problem of windows has been inferred from the human perspective rather than bird vision and flight behavior [6]. Specifically, a flying bird understands the world via acute lateralized vision, optic flow fields, and head movements, whereas a human perceives the external environment with highly acute binocular vision in the frontal space. Martin [6] argues a sensory ecology approach that emphasizes bird vision and flight behavior may yield the most fruitful understanding of why birds collide with structures. This has been applied to species vulnerable to collisions with other obstacles, such as power lines, and future work should examine avian sensory ecology in reference to sheet glass in urban systems.

Although BWCs depended on development and window area, the relationships resembled space-filling distributions (Fig. 3). These patterns arise when the predictor variable sets an upper limit on the response variable and other factors are likely important at certain levels of the predictor variables [44]. For example, fatalities were infrequent at low window area, and no fatalities were observed below a threshold window area of 22 m^2^. However, when window area was high, the number of fatalities was variable. Development also set an upper limit on BWCs. Collisions generally decreased as development increased, but there was wide variation in the number of fatalities when development was low. BWCs were also not observed at sites constructed in >66% impervious surfaces, suggesting that birds are at low risk of collisions at buildings in high development. Although window area and development set upper bounds on BWCs, other factors, such as degree of reflectivity and tinting of windows may explain additional variation in fatalities. There was a range of window types in study buildings that included clear panes in small residential structures and highly reflective and tinted glass in commercial buildings. Limited research suggests no differences in BWCs between observer-defined clear and reflective glass panes [22]. However, controlled experiments are needed to clarify the role of window treatment on collisions.

Site-specific comparisons between birds observed during point counts and those documented as carcasses suggest that most species never die from window collisions. The small percentage of birds found as carcasses included the American Robin, Cedar Waxwing, and White-throated Sparrow, which ranged from low to high relative abundance. Species recorded as fatalities have also been documented in local studies [5], [18]–[20], [22]–[23] and are common in urban areas. This similarity may be explained via faunal homogenization where urban-adapted bird species among established urban landscapes converge at the continental scale [2], [36]–[49]. If so, the effects of windows on population persistence in these species warrants further investigation, especially as this relates to higher mortality in juveniles than adults [50].

Presence of feeder stations at study buildings correlated positively with relative abundance, which is consistent with bird communities in other urban areas [17]. However, feeders at study buildings did not influence BWCs, which is consistent with Dunn [18] who found that 91% of 5,500 houses with feeders had no mortality from window strikes. Moreover, ∼20% of 995 fatalities reported by Dunn [18] were focused at just 8 residences in areas of low development. Houses constructed in exurban areas, i.e., ‘rural development’, contain almost no adjacent impervious surfaces, and our results suggest that only modest levels of window area in this environmental context will result in relatively high BWCs. House Sparrows were one of the most abundant species at study buildings with feeders, but were not documented as a collision fatality, which Dunn [18] also found. Interestingly, invasive populations of this species exclude up to 30% of other urban species [37]. Thus, high abundances of House Sparrows might aid in reducing collision risk at structures by inhibiting the presence of vulnerable species.

Overall, our results suggest that mortality resulting from window collisions is an important conservation issue at buildings with high window area and constructed in areas of low development. As landscapes become increasingly developed, it will be important to continue to evaluate the magnitude and patterns of BWCs and assess how urban populations respond to this source of mortality. Future studies should employ experimental designs that account for biases known to affect detection probability of carcasses. We are unaware of studies that have assessed the fate of birds that are not immediately fatally injured following a window strike [22], which is another form of bias leading to imperfect detection. Research is needed on how BWCs compare to other anthropogenic threats and whether multiple threats interact to affect bird populations, as has been shown for some amphibians [52]. Birds tracked through the urban landscape via radio telemetry are known to die more from predation by cats, disease, and vehicle collisions than from BWCs [53]–[56]. However, the localized nature of BWCs suggests that this threat is context-dependent, and studies should address how both environmental and structural factors drive variation in mortality.

### Conservation Implications

Current estimates of BWCs assert a modest level of within-site variation in mortality (1–10 fatalities/building/year) and this range was used to extrapolate an overall estimate of fatalities to all existing buildings in the United States, which implies little to no variation in BWCs among all buildings [7]. Interest groups and municipalities primarily use these estimates for planning and implementation of preventative measures, e.g., the recently passed ‘Standards for Bird-safe Buildings’ in San Francisco, California, USA [57]. Because overall mortality estimates for broad geographic scales fail to convey spatial variation in mortality, relatively equal conservation efforts end up being applied among sites of unequal numbers of fatalities. That is, hotspot areas with excessive collision mortality may receive insufficient resources to reduce the effects of mortality, and unneeded resources may be applied to sites with little to no mortality. These implications call into question the practical uses of overall imprecise mortality estimates and prompt the need for a stronger emphasis on understanding spatial variation in BWCs.

We demonstrated how one might use factors known to influence window collisions in modeling variation in risk for a given landscape. For proposed development, urban planners could minimize future collision mortality by mapping proportion of impervious development for the landscape, and identify areas of high development, e.g., >66% impervious surfaces, in which to construct buildings. Mapping predicted fatalities for the landscape can be a powerful tool in evaluating risk and making informed decisions about where to focus resources aimed at prevention. For example, wildlife managers could focus prevention measures aimed at minimizing collisions at high-risk sites, which appear to cause relatively high mortality and affect species of conservation concern (e.g., many species of long-distance migrating wood warblers). Therefore, modeling spatial variation in collision mortality derived from data-driven experimental designs would improve evaluations of population impacts and allow conservation resources to be applied in a triage manner by implementing effective preventative measures in the most pressing settings [50].

In light of our results, land use legacies and the nature of urban growth, such as transient dynamics [13], across broad geographic scales should affect variation within and among landscapes in the numbers of individuals and species impacted by window collisions. We currently have a biased understanding of the spatial and temporal aspects of BWCs since previous research has been confined to large commercial and high-rise buildings [4]–[50]. At a broader scale, study sites have generally been located in areas important for bird migration, such as along major migratory pathways and at urban sites at the edges of large bodies of water where staging areas (migration stopover locations) concentrate migrants [11]–[19], [20]–[22], [23]–[51], this study. These studies report that window collisions disproportionally affect short- and long-distance migrant species and occur more during spring and fall migration than in summer and winter. Indeed, the cities in which these studies have been conducted include not just skyscrapers, but also a range of building sizes and vast areas of residential development [24]. Future studies should focus on how the pattern and magnitude of BWCs among urban areas reflect landscape structure and functional connectivity as was recently demonstrated for avian mortality at communication towers [58]. For example, large cities settled along migratory paths should display high variation in BWCs across the landscape, whereas low variation in BWCs would be expected at villages consisting of only small buildings (i.e., low window area) outside of migratory routes.

## Supporting Information

Figure S1 **Relationship between carcass observability and mean (±1 SE) detection probability of carcasses for two field workers at 20 buildings.**
(PDF)

Table S1 **Most-supported models of carcass detection probability at 20 buildings in an urban landscape in Illinois, USA, 2010.**
(PDF)

Table S2 **Seasonal sampling structure that corresponded to migration strategy and timing of breeding in Illinois, USA, 2010.**
(PDF)

Table S3 **List of study buildings, land cover categories, window area, development, presence of feeder stations, and number of carcasses documented and predicted in Illinois, USA, 2010.**
(PDF)

Table S4 **Maximum number of individuals observed for each species during point count surveys and total carcasses (shaded and in parentheses) resulting from window collisions at each study building and season in Illinois, USA, 2010.**
(PDF)

Text S1 **Analysis of detection probability.**
(PDF)
