# Supplementary material for: Window Area and Development Drive Spatial Variation in Bird-Window Collisions in an Urban Landscape
Source: PLoS One. 2013 Jan 9;8(1):e53371. doi: 10.1371/journal.pone.0053371 (PMC3541239; doi:10.1371/journal.pone.0053371)

Figure S1. Relationship between carcass observability and mean (±1 SE) detection probability of carcasses for two field workers at 20 buildings.

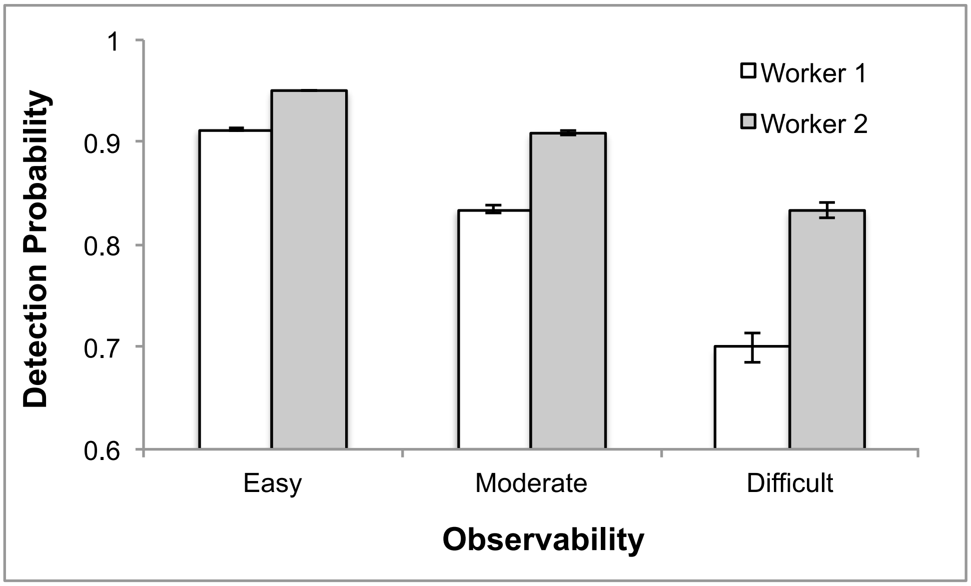

Supplement: Figure S1 — Relationship between carcass observability and mean (±1 SE) detection probability of carcasses for two field workers at 20 buildings. (PDF) [file pone.0053371.s001.pdf]
