# Supplementary material for: Window Area and Development Drive Spatial Variation in Bird-Window Collisions in an Urban Landscape
Source: PLoS One. 2013 Jan 9;8(1):e53371. doi: 10.1371/journal.pone.0053371 (PMC3541239; doi:10.1371/journal.pone.0053371)
Supplement: Table S1 — Most-supported models of carcass detection probability at 20 buildings in an urban landscape in Illinois, USA, 2010. (PDF) [file pone.0053371.s002.pdf]

Table S1. Most-supported models of carcass detection probability at 20 buildings in an urban landscape in Illinois, USA, 2010.

Model	ΔAIC_C	ω_i	$-2l$	K
O	0.00	0.30	80.18	3
O + W	0.04	0.29	77.05	4
O + W + O*W	2.70	0.08	76.09	5
W	2.77	0.07	82.95	3
O + N	2.80	0.07	79.81	4
Intercepts	2.98	0.07	85.95	2
O + W + N	3.50	0.05	76.89	5
N + W + N*W	4.88	0.03	78.27	5
N	5.23	0.02	85.41	3
N + W	5.38	0.02	82.39	4

Main effects include carcass observability (O), individual field worker (W), and survey order (N). Summary includes the relative difference between model AIC_C and the best model (ΔAIC_C), Akaike weights (ω_i), twice the negative log-likelihood ($-2l$), and number of parameters estimated (K). The overall model-averaged detection probability was 0.88 (SE = 0.01). Detection probability was related to carcass observability and field worker (see Fig. S1).
