# Supplementary material for: Window Area and Development Drive Spatial Variation in Bird-Window Collisions in an Urban Landscape
Source: PLoS One. 2013 Jan 9;8(1):e53371. doi: 10.1371/journal.pone.0053371 (PMC3541239; doi:10.1371/journal.pone.0053371)
Supplement: Table S2 — Seasonal sampling structure that corresponded to migration strategy and timing of breeding in Illinois, USA, 2010. (PDF) [file pone.0053371.s003.pdf]

Table S2. Seasonal sampling structure that corresponded to migration strategy and timing of breeding in Illinois, USA, 2010.

	Beginning Date of	Permanent Residents		Short-distance Migrants			Long-distance Migrants[b]	
Season	Survey Period[a]	Non-breeding	Breeding	Non-breeding	Migrating	Breeding	Migrating	Breeding
Winter	8 Jan	X		X				
	22 Jan[c]	X		X				
	5 Feb[d]	X		X				
Spring	21 Mar		X	X	X			
	18 Apr		X		X	X	X	
	10 May[c]		X		X	X	X	
Summer	16 Jun[e]		X			X		X
	5 Jul		X			X		X
	22 Jul		X			X		X
Fall	12 Sep		X			X	X	
	26 Sep		X		X		X	
	17 Oct	X			X		X	

Three point count surveys and daily carcass searches were conducted at 20 study buildings during each survey period, which lasted seven days.

[a]Point count surveys were completed only during favorable weather conditions (Ralph et al. 1995)
[b]Non-breeding column is not included since these species do not spend the winter in this region
[c]Inclement weather permitted only two point count surveys during this week
[d]Carcass surveys on 9 February were postponed to 13 February due to inclement weather
[e]Searcher efficiency was evaluated

References for Table S2

Ralph CJ, Sauer JR, Droege S (1995) Monitoring bird populations by point counts. Albany: U.S. Department of Agriculture, Forest Service General Technical Report PSW-149.
