# Supplementary material for: Window Area and Development Drive Spatial Variation in Bird-Window Collisions in an Urban Landscape
Source: PLoS One. 2013 Jan 9;8(1):e53371. doi: 10.1371/journal.pone.0053371 (PMC3541239; doi:10.1371/journal.pone.0053371)
Supplement: Table S3 — List of study buildings, land cover categories, window area, development, presence of feeder stations, and number of carcasses documented and predicted in Illinois, USA, 2010. (PDF) [file pone.0053371.s004.pdf]

Table S3. List of study buildings, land cover categories, window area, development, presence of feeder stations, and number of carcasses documented and predicted in Illinois, USA, 2010.

				Top Model			Surrogate Model		
Study Building	Land Cover Category[a]	Minimum Distance to Feeder Station (m)	Total Carcasses	Window Area (m^2)	Digitized Development[b]	Predicted Annual Fatalities[c]	Floorspace[d] (m^2)	NLCD Develoment[e]	Predicted Annual Fatalities[f]
1	Low/medium Urban Density	46	0	8	0.38	0.7	128	0.46	0.9
4	Forested Land	-	2	26	0.30	2.7	623	0.28	7.4
5	Forested Land	1	5	72	0.18	10.5	392	0.22	6.9
6	Low/medium Urban Density	5	0	17	0.33	1.7	109	0.42	1.0
9	Low/medium Urban Density	9	0	16	0.28	1.9	177	0.35	2.0
10	High Urban Density	-	0	18	0.82	0.2	425	0.86	0.4
11	Forested Land	14	1	34	0.36	2.6	321	0.39	2.7
12	Urban Open Space	1	1	24	0.10	5.8	120	0.12	4.3
13	Urban Open Space	-	1	38	0.39	2.5	694	0.50	3.0
15	Low/medium Urban Density	46	0	13	0.43	0.9	111	0.44	0.9
16	Urban Open Space	-	2	135	0.23	14.6	402	0.45	2.5
17	High Urban Density	-	0	21	0.79	0.3	149	0.92	0.1
18	High Urban Density	-	0	26	0.66	0.6	333	0.75	0.5
19	Low/medium Urban Density	-	0	41	0.41	2.5	235	0.43	1.8
20	Forested Land	7	0	14	0.36	1.3	118	0.40	1.2
21	Forested Land	-	11	516	0.19	52.1	3,746	0.26	33.6
22	Urban Open Space	-	3	298	0.56	6.7	2,357	0.33	17.3
23	High Urban Density	-	6	1,876	0.66	19.6	14,775	0.71	12.7
24	High Urban Density	-	0	166	0.62	3.2	1,229	0.65	2.3
25	Urban Open Space	-	2	795	0.53	16.9	6,614	0.33	38.4

[a]Land cover categories from the 1999-2000 Illinois Land Cover Classification map used to select study sites (Illinois Department of Agriculture 2009).
[b]Development was digitized within a 50-m buffer of a study building's edge.
[c]Calculated from the most supported model of collisions (Table 3) using beta coefficients: predicted fatalities = EXP(-3.2772 + 0.8316*ln(Window Area)-4.3154*Development)*17.33.
[d]Floorspace for single story buildings (#'s 4, 10, and 16) not available through the Rock Island County GIS department and rather was measured in ArcMap from digitized building footprints; floorspace for multistory Building 23 was calculated from the regression of known floorspace and window area of the remaining study buildings ($r^2 = 0.98$; $y = 7.867x + 19.316$).
[e]Measured within a 50m buffer around each model building footprint using the National Land Cover Database (NLCD) 2006 Percent Developed Imperviousness Proportion (Fry et al. 2011).
[f]Calculated from a surrogate model of collisions using beta coefficients: predicted fatalities = EXP(-4.5946+0.7853*ln(Floorspace)-4.6185*Development)*17.33.

References for Table S3

Fry J, Xian G, Jin S, Dewitz J, Homer C, et al. (2011) Completion of the 2006 National Land Cover Database for the Conterminous United States. PE&RS 77(9): 858-864.

Illinois Department of Agriculture (2009) Land cover data for Illinois, 1999–2000. Available: http://www.isgs.illinois.edu/nsdihome/webdocs/landcover/landcover99–00.html. Accessed 2009 Aug 21.
