# Supplementary material for: Window Area and Development Drive Spatial Variation in Bird-Window Collisions in an Urban Landscape
Source: PLoS One. 2013 Jan 9;8(1):e53371. doi: 10.1371/journal.pone.0053371 (PMC3541239; doi:10.1371/journal.pone.0053371)
Supplement: Table S4 — Maximum number of individuals observed for each species during point count surveys and total carcasses (shaded and in parentheses) resulting from window collisions at each study building and season in Illinois, USA, 2010. (PDF) [file pone.0053371.s005.pdf]

Table S4. Maximum number of individuals observed for each species during point count surveys and total carcasses (shaded and in parentheses) resulting from window collisions at each study building and season in Illinois, USA, 2010. Total species observed for each season: Winter = 23, Spring = 57, Summer = 38, Fall = 49.

			Study Building																			
Season	Common Name	Scientific Name[a]	1	4	5	6	9	10	11	12	13	15	16	17	18	19	20	21	22	23	24	25
Winter	Rock Pigeon	*Columba livia*				6									15		2		10			
	Mourning Dove	*Zenaida macroura*				2	3		1								3					
	Red-bellied Woodpecker	*Melanerpes carolinus*	1	1	2	2				2	1	1					2	2	1			2
	Downy Woodpecker	*Picoides pubescens*	2	1	3	1	1		3	3	2	1	2				2	4				3
	Hairy Woodpecker	*Picoides villosus*			2																	
	Blue Jay	*Cyanocitta cristata*				2	1			2		2	2				1	1	3			3
	American Crow	*Corvus brachyrhynchos*	3									4										
	Black-capped Chickadee	*Poecile atricapillus*	4	6	7	2	5		6	5	7	4				4	3	9	6			6
	Tufted Titmouse	*Baeolophus bicolor*								1	3											
	White-breasted Nuthatch	*Sitta carolinensis*		2	3					3	4	1					2	5	2			4
	Brown Creeper	*Certhia americana*			2													2				
	Carolina Wren	*Thryothorus ludovicianus*								1								2	2			
	European Starling	*Sturnus vulgaris*	3		2	10	3	1		3		5	5	1	7	3	5	2			12	1
	American Tree Sparrow	*Spizella arborea*		2														3	4			
	Fox Sparrow	*Passerella iliaca*																	1			
	Song Sparrow	*Melospiza melodia*											1									
	White-throated Sparrow	*Zonotrichia albicollis*																2				
	Dark-eyed Junco	*Junco hyemalis*	2	5	4		6		3	8	4		1			6	3	15 (1)	11			3
	Northern Cardinal	*Cardinalis cardinalis*	3	2	4	2	4		3	8	3	2	2		2	9	1	5 (1)	8	1	2	6
	Brown-headed Cowbird	*Molothrus ater*	12																			
	House Finch	*Carpodacus mexicanus*				2	5		1	3			2			6		2	1		4	
	American Goldfinch	*Spinus tristis*	5		16		2		1	2	11	3	1				7	10	9			4
	House Sparrow	*Passer domesticus*	33	2	14	39	24		6	20	1	8	4		6	7	20	4	4		16	12
Spring	Killdeer	*Charadrius vociferus*						4	1					2	2							
	Rock Pigeon	*Columba livia*	3										4	3	7	4				3		
	Mourning Dove	*Zenaida macroura*	3	6	3	6	8		5	4	2	1	5		2	4		5	5	6	3	2
	Chimney Swift	*Chaetura pelagica*										3			2	3		1				
	Red-headed Woodpecker	*Melanerpes erythrocephalus*		2	1																	1
	Red-bellied Woodpecker	*Melanerpes carolinus*	1	2	3	1	2		3	3	3						2	2	3			1
	Downy Woodpecker	*Picoides pubescens*	3	3	3	1	2		3	2		2	1			1	2	5	3			1
	Hairy Woodpecker	*Picoides villosus*		1						1									1			
	Northern Flicker	*Colaptes auratus*		3	1		1			3								1	2			2
	Least Flycatcher	*Empidonax minimus*																				1
	Eastern Phoebe	*Sayornis phoebe*		2	3		1		2	1	1											1
	Great Crested Flycatcher	*Myiarchus crinitus*																3				
	Red-eyed Vireo	*Vireo olivaceus*								1									1			1
	Blue Jay	*Cyanocitta cristata*		2	2	2	5		3	7		1	1				3	6	4			2
	American Crow	*Corvus brachyrhynchos*								3	2	3				3		2			2	3
	Northern Rough-winged Swallow	*Stelgidopteryx serripennis*											2							2		
	Tree Swallow	*Tachycineta bicolor*																		6		
	Bank Swallow	*Riparia riparia*												5						10		
	Barn Swallow	*Hirundo rustica*												6						10		
	Cliff Swallow	*Petrochelidon pyrrhonota*												2								
	Black-capped Chickadee	*Poecile atricapillus*	3	3	3	2	2		4	4	3	3	3			2	2	6	4			7
	Tufted Titmouse	*Baeolophus bicolor*			5		1				1						2	3	2			1
	White-breasted Nuthatch	*Sitta carolinensis*		3	3	1	3			1	1	1	2				2	2	2			2
	Carolina Wren	*Thryothorus ludovicianus*			2				1	2								2				2
	House Wren	*Troglodytes aedon*	4	3	2		2		2	2	3	2					2	4			2	2
	Blue-gray Gnatcatcher	*Polioptila caerulea*									2							1				1
	Ruby-crowned Kinglet	*Regulus calendula*		2	3				1	5	2					1	1	2				2

	American Robin	*Turdus migratorius*	10	12	11	12	10	8	9	10	11	7	12		8	9	5	9	5	8	6	6
	Swainson's Thrush[b]	*Catharus ustulatus*											(1)									
	Gray Catbird[b]	*Dumetella carolinensis*			2		2			5	4		2				1	6				1 (1)
	European Starling	*Sturnus vulgaris*	10	6	2	12	9	9	2	4	6	2	9	7	10	4	7	2	3	15	8	3
	Cedar Waxwing	*Bombycilla cedrorum*		2				11		4	2							3				2
	Black-and-white Warbler	*Mniotilta varia*																	1			
	Tennessee Warbler	*Oreothlypis peregrina*		4	8	1	1			4	3	4	3				4	3	3		2	4
	Orange-crowned Warbler	*Oreothlypis celata*		1																		
	Nashville Warbler	*Oreothlypis ruficapilla*		4	1					2	1		2					2	3			
	Common Yellowthroat	*Geothlypis trichas*								1												
	American Redstart	*Setophaga ruticilla*																	1			
	Chestnut-sided Warbler	*Setophaga pensylvanica*		1								3							1			
	Palm Warbler	*Setophaga palmarum*		2					2	2			1				1	3	1			
	Yellow-rumped Warbler	*Setophaga coronata*		6	4				6	4			2				2	6				7
	Eastern Towhee	*Sayornis phoebe*									1											
	Chipping Sparrow	*Spizella passerina*	2	4	3	4	3	1	6	4	1	3	4		1	2	5	2	4		4	3
	Song Sparrow	*Melospiza melodia*						1		2	3		4		3			2	2	4	3	2
	White-throated Sparrow	*Zonotrichia albicollis*	1	4	5		1		2	8	3							8	4			3
	White-crowned Sparrow	*Zonotrichia leucophrys*					1			2												
	Dark-eyed Junco	*Junco hyemalis*	7		8	3			10	11	3		5			4	13	13	2		2	3
	Northern Cardinal	*Cardinalis cardinalis*	3	4	9	6	5		4	10	7	4	2		2	5	5	8	6	2	4	9
	Rose-breasted Grosbeak	*Pheucticus ludovicianus*		1							1						1					
	Indigo Bunting	*Passerina cyanea*		4						3			2					5	4 (1)			2
	Red-winged Blackbird	*Agelaius phoeniceus*	3	2		3	2		6	5	2		10	8	5	1		6	5	6	2	
	Common Grackle	*Quiscalus quiscula*	11		2	14			3	3			9	4	9	6		2	2	12	1	
	Brown-headed Cowbird	*Molothrus ater*	4	4	4	3	5		5	2	4	6	7		1	8	2	6	6	7	5	3
	Baltimore Oriole	*Icterus galbula*		2							1		1						2			
	House Finch	*Carpodacus mexicanus*	6	3	3	1	5		2	5	6	5	4		4	4	1	2	1	2	8	2
	American Goldfinch	*Spinus tristis*	7	4	4	5	6	1	6	6	4	6	7	2		4	4	9	7	5	3	4
	House Sparrow	*Passer domesticus*	48	4	5	20	23	2	8	9	7	9	8		11	8	27	5	6		34	9
Summer	Killdeer	*Charadrius vociferus*												3								
	Ring-billed Gull	*Larus delawarensis*						14														
	Rock Pigeon	*Columba livia*				8									3	3						
	Mourning Dove	*Zenaida macroura*	3	3	3	3	4	3	3	2		2	6	2	15	4	2	3	3	16 (1)	5	
	Chimney Swift	*Chaetura pelagica*	2		1	3	2		1			5		2	4	7	4		1	5	3	2
	Ruby-throated Hummingbird	*Archilochus colubris*			1												1					
	Red-headed Woodpecker	*Melanerpes erythrocephalus*		1																		
	Red-bellied Woodpecker	*Melanerpes carolinus*		2	2					2							2	3	1			
	Downy Woodpecker[b]	*Picoides pubescens*	1	2	2	1	1		1	4	2	1	1			1	3	2	(1)			2
	Northern Flicker	*Colaptes auratus*		2									2					3				
	Eastern Wood-Pewee	*Contopus virens*		2	2												5	2	1			
	Eastern Phoebe	*Sayornis phoebe*		6	2					2												
	Great Crested Flycatcher	*Myiarchus crinitus*								1												
	Blue Jay	*Cyanocitta cristata*		1	2				2	4		1				2	1	3	1			
	American Crow	*Corvus brachyrhynchos*										1										
	Barn Swallow	*Hirundo rustica*						1						5								
	Black-capped Chickadee	*Poecile atricapillus*	1	6	7	1	7		4	5	4	5	2			5	5	6	1			2
	Tufted Titmouse	*Baeolophus bicolor*			2		1											1				
	White-breasted Nuthatch	*Sitta carolinensis*		1	2	5			1	2	2	1					2	3				2
	Carolina Wren	*Thryothorus ludovicianus*		2														1				
	House Wren	*Troglodytes aedon*	2	4	5	3	3		4	4	6	3	2			2	3	4	2			3
	Blue-gray Gnatcatcher	*Polioptila caerulea*		2							2		1					1				2
	Eastern Bluebird	*Sialia sialis*								1												
	American Robin	*Turdus migratorius*	7	13	5 (1)	7	9	3	15	16	5	3	9		4	9	6	14	10	7 (1)	3	5
	Gray Catbird	*Dumetella carolinensis*		2	3	1	2		6	3	4		4			4		4	4		2	3

	European Starling	*Sturnus vulgaris*	4	3		7	4	11	5			2	12	6	11	2	1		4	5	5	
	Cedar Waxwing	*Bombycilla cedrorum*	2	2		2	6		3			5	7	5	2				4	2 (1)	2	
	Chipping Sparrow	*Spizella passerina*	3	4	3	4	3		5	3	2	4	5			2		2	4		4	
	Song Sparrow	*Melospiza melodia*						1			2		3	1	2				1	2	1	
	Northern Cardinal	*Cardinalis cardinalis*	2	7	6 (1)	3	4		4	5	5	7	2		2	4	6	6	6	1	2	5
	Indigo Bunting	*Passerina cyanea*		2					2	1	2							1				1
	Red-winged Blackbird	*Agelaius phoeniceus*		1				4					9	1	2				3	4		
	Common Grackle	*Quiscalus quiscula*	3	1		9	3		2	2			6	3	9	3		1	4 (1)	14 (1)	3	
	Brown-headed Cowbird	*Molothrus ater*	2	3			2		2	4		1										
	Baltimore Oriole	*Icterus galbula*		1						1												
	House Finch	*Carpodacus mexicanus*	3		3		2		4	3		2	6			2		2	2	1	4	
	American Goldfinch	*Spinus tristis*	3	5	8	6	5		7	7	9	5	10	1	6	5	6	3	9	8	7	7
	House Sparrow	*Passer domesticus*	45	4	10	23	17	3	9	22	2	26	16		7	3	19	7	4		26	3
Fall	Killdeer	*Charadrius vociferus*						1						4								
	Ring-billed Gull	*Larus delawarensis*						1						11						4		
	Herring Gull	*Larus argentatus*												8								
	Rock Pigeon	*Columba livia*				10		17						3	10	3						
	Mourning Dove	*Zenaida macroura*	2			4	1		1				10	4	7		3				2	
	Chimney Swift	*Chaetura pelagica*				6	5			2		4			5	4	3			6	3	4
	Red-bellied Woodpecker	*Melanerpes carolinus*	2	2	2	2	1			2	1	2	2			1	4	3	1			2
	Yellow-bellied Sapsucker[b]	*Sphyrapicus varius*	1	2						2								(1)				
	Downy Woodpecker	*Picoides pubescens*		3	2	2			1	2	1	3	2				4	2	2		1	1
	Hairy Woodpecker	*Picoides villosus*		2								2					1					1
	Northern Flicker	*Colaptes auratus*		4					5	3		4	1			1		1			1	
	Eastern Wood-Pewee	*Contopus virens*		2									1									
	Eastern Phoebe	*Sayornis phoebe*								1												
	Warbling Vireo	*Vireo gilvus*		1																		
	Red-eyed Vireo	*Vireo olivaceus*		2																		
	Blue Jay	*Cyanocitta cristata*	2	5	3	14	5		3	7	3	9	3		2	2	3	4 (1)	5		5	4
	American Crow	*Corvus brachyrhynchos*	2								1	5				1		3				6
	Black-capped Chickadee	*Poecile atricapillus*	4	5	7	4	5	3	6	6	4	8	3			4	6	8	4	1	1	3
	Tufted Titmouse	*Baeolophus bicolor*																2				
	Red-breasted Nuthatch	*Sitta canadensis*								1		2										
	White-breasted Nuthatch	*Sitta carolinensis*	1	3	2	2	1		3	2	2	2	1			1	4	3	2			1
	Carolina Wren	*Thryothorus ludovicianus*														1						2
	House Wren	*Troglodytes aedon*											3					1				
	Golden-crowned Kinglet	*Regulus satrapa*		5			3										2	3				
	Ruby-crowned Kinglet	*Regulus calendula*		2												1						
	Gray-cheeked Thrush	*Catharus minimus*																1				
	Swainson's Thrush	*Catharus ustulatus*			1				1													
	Hermit Thrush[b]	*Catharus guttatus*			(2)																	
	American Robin	*Turdus migratorius*	14	13 (1)	6	14	3	2	12	14	10	5	4		1	10	7	2	15	2	15	7
	Gray Catbird	*Dumetella carolinensis*					3			4	2					1			3			4
	European Starling	*Sturnus vulgaris*	3	2 (1)	2	5	5	11	4	8	9	9	11	12	10	10	13	2	2	3	12	10
	Cedar Waxwing	*Bombycilla cedrorum*	3	11	4	9	11		10	11	3	2	8			4		3 (3)	7	1	6	3
	Tennessee Warbler	*Oreothlypis peregrina*		1																		
	Nashville Warbler	*Oreothlypis ruficapilla*		3																		
	Common Yellowthroat[b]	*Geothlypis trichas*									(1)											
	Yellow-rumped Warbler	*Setophaga coronata*					2			3	3		2					2	3			1
	Chipping Sparrow	*Spizella passerina*	2	6			3			2	2	2	6		1	6			1		1	
	Song Sparrow	*Melospiza melodia*		3						2			7									
	Swamp Sparrow	*Melospiza georgiana*								1												
	White-throated Sparrow	*Zonotrichia albicollis*		13	3		7		5 (1)	5 (1)	3	1	4					13	7			2
	Dark-eyed Junco	*Junco hyemalis*	1	3	6	3	2		7	7		3	8			4	5	7	9		13	1
	Northern Cardinal	*Cardinalis cardinalis*	4	2	3	3	4		5	5	4	5	4			4	3	6	6	3	2	5

Rose-breasted Grosbeak	*Pheucticus ludovicianus*					2											1				
Red-winged Blackbird	*Agelaius phoeniceus*	11	5			9		11	3	12							8	4		7	10
Common Grackle	*Quiscalus quiscula*	2	3	2	3	3	2	2	4	3	3			8	8	5	2	7		4	5
Brown-headed Cowbird	*Molothrus ater*	2						2													
House Finch	*Carpodacus mexicanus*	2		4	6	3	3	4	6		5	7			2	5	1			1	
American Goldfinch	*Spinus tristis*	4	9	6	4	10	2	3	6	5	2	10			4	3	4	4	1	4	3
House Sparrow	*Passer domesticus*	40	7	10	38	39	10	10	8	7	12	23		7	8	17	8	1		22	6

[a]Scientifc names follow The American Ornithologists' Union (2012) Checklist of North American birds. Available: http://www.aou.org/checklist/north/full.php. Accessed: 2012 Jun 10.

[b]Observed as a carcass but not during point count surveys at respective buildings.
