# Supplementary material for: Window Area and Development Drive Spatial Variation in Bird-Window Collisions in an Urban Landscape
Source: PLoS One. 2013 Jan 9;8(1):e53371. doi: 10.1371/journal.pone.0053371 (PMC3541239; doi:10.1371/journal.pone.0053371)
Supplement: Text S1 — Analysis of detection probability. (PDF) [file pone.0053371.s006.pdf]

Text S1. Analysis of detection probability.

We evaluated detection probability for two field observers during the first survey-week in summer (Table S2). Bird carcasses were planted at all buildings for six consecutive days to simulate fatalities from BWCs. Each of the two observers surveyed buildings for three consecutive days. Carcass plantings were unannounced to field workers (Gehring et al. 2009). Carcasses were salvaged previously from below windows at Augustana College, Rock Island, Illinois, which is located in the same urban area. Species of carcasses (N = 15) ranged in size from a Brown Creeper (*Certhia americana*; 8 g) to a Mourning Dove (*Zenaida macroura*; 120 g) (Sibley 2000) and corresponded to the same species found in the study area and potentially killed at study buildings. Bird carcasses were collected from July 2007 to May 2010, frozen immediately after collection, and allowed to thaw before placement at a building. Whole carcasses were used because fatalities from window strikes rarely display external injuries, and this is what field workers would naturally encounter.

For each building-day combination, we randomized bird species and location of carcasses with respect to the cardinal direction of one of four walls. Carcass placement among various substrates was roughly equal for each day and field worker. Carcasses were placed below a window simulating the appearance and location of a bird following a window collision, thus birds were on their backs and ~1 m from windows.

Carcass detection is influenced by body size, color, visibility in the environment, and performance of field workers, e.g., search image, motivation, and fatigue (Ward et al. 2006, Smallwood et al. 2010, Huso 2011). Detection probability in the summer likely represents a reasonable estimate of carcass detection in the fall because vegetation cover was similar between the two seasons (S. B. Hager, unpublished data). Detection estimates in the summer are also conservative estimates for winter and spring when visibility is high due to low vegetation cover.

We used occupancy modeling and program PRESENCE (v. 3.1) to calculate detection probability for carcasses at each study building (MacKenzie et al. 2006). We built single-season models of detection probability that were based on repeated surveys for carcasses. A repeated survey consisted of one trip around a building on a single day. Occupancy probability was fixed at 1.0 for each site because all sites were "occupied" by carcasses. We built models with different combinations of covariates to model heterogeneity in detection probability among sites. Covariates included surveyor (n = 2), bird observability, and survey order. Observability represented difficulty in observing carcasses and was based on plumage color (dark = difficult to observe; light = easy to observe) and substrate. The main substrates encountered were grass, stone ground cover, hardwood deck/sidewalk, low-laying woody ground cover, and hardwood landscaping mulch. We used an ordinal rank to record observability for each bird (1 = easily detectable, 2= moderately detectable, 3 = difficult to detect). For example, a Mourning Dove carcass on a sidewalk would be ranked as "1", whereas a Brown Creeper in hardwood mulch ground cover would be "3". Survey Order represented the chronological order of surveys completed by a worker each day, and it was used to test for temporal trends in detection during an individual survey day. We included interaction effects between observer and both bird observability and survey order. We used the Akaike Information Criterion corrected for small sample size (AICc) to rank the support

of models that included different combinations of covariates. Point estimates of detection probability were model-averaged using to entire model set.

References for Text S1

Gehring J, Kerlinger P, Manville AM II (2009) Communication towers, lights, and birds: successful methods of reducing the frequency of avian collisions. Ecol Applications 19: 505–514.

Huso MMP (2011) An estimator of wildlife fatality from observed carcasses. Envirometrics 22: 318-329.

MacKenzie DI, Nichols JD, Royle JA, Pollock KH, Bailey LA, et al. (2006) Occupancy modeling and estimation. San Diego, California: Elsevier. 344 p.

Sibley DA (2000) The Sibley guide to birds. Knopf: New York. 544 p.

Smallwood KS, Bell DA, Snyder SA, Didonato JE (2010) Novel scavenger removal trials increase wind turbine-caused avian fatality estimates. J Wild Manage 74: 1089-1097.

Ward MR, Stallknecht DE, Willis J, Conroy MJ, Davidson WR (2006) Wild bird mortality and West Nile virus surveillance: biases associated with detection, reporting, and carcass persistence. J Wild Dis 42: 92-106.
